# One-Pot Synthesis of LiFePO_4_/N-Doped C Composite Cathodes for Li-ion Batteries

**DOI:** 10.3390/ma15144738

**Published:** 2022-07-06

**Authors:** Baoquan Zhang, Shuzhong Wang, Lu Liu, Yanhui Li, Jianqiao Yang

**Affiliations:** Key Laboratory of Thermo-Fluid Science and Engineering of Ministry of Education, School of Energy and Power Engineering, Xi’an Jiaotong University, 28 Xianning West Road, Xi’an 710049, China; bqzhang1017@outlook.com (B.Z.); 15162138902@163.com (L.L.); yhli19@mail.xjtu.edu.cn (Y.L.)

**Keywords:** lithium-ion batteries, LiFePO_4_, N-doped, core–shell structure

## Abstract

LiFePO_4_/N-doped C composites with core–shell structures were synthesized by a convenient solvothermal method. Cetyltrimethylammonium bromide (CTAB) and glucose were used as nitrogen and carbon sources, respectively. The growth of LiFePO_4_ nanocrystals was regulated by CTAB, resulting in an average particle size of 143 nm for the LiFePO_4_/N-doped C. The N atoms existed in the carbon of LiFePO_4_/N-doped C in the form of pyridinic N and graphitic N. The LiFePO_4_/N-doped C composites delivered discharge specific capacities of 160.7 mAh·g^−1^ (0.1 C), 128.4 mAh·g^−1^ (5 C), and 115.8 mAh·g^−1^ (10 C). Meanwhile, no capacity attenuation was found after 100 electrochemical cycles at 1 C. N-doping enhanced the capacity performance of the LiFePO_4_/C cathode, while the core–shell structure enhanced the cycle performance of the cathode. The electrochemical test data showed a synergistic effect between N-doping and core–shell structure on the enhancement of the electrochemical performance of the LiFePO_4_/C cathode.

## 1. Introduction

The fossil energy that the modern economy depends on is gradually being exhausted, and the new energy industry has become a strategic platform for global economic development. The demand for new energy has promoted the vigorous development of energy storage devices. Lithium-ion batteries (LIBs) [1,2], electrochemical capacitors [3,4], lithium-sulfur batteries [5,6,7] are being developed. LIBs have been commercialized due to their high energy density and long cycle life. Lithium iron phosphate (LiFePO_4_), as one of the LIBs cathode materials, has good thermal stability, excellent environmental compatibility, and low cost. It is one of the most popular cathode materials used in fixed energy storage equipment and electric vehicles (EVs) [8,9,10]. However, the discontinuous FeO_6_ co-edge octahedral network in LiFePO_4_ crystals and the PO_4_ tetrahedrons between them affect the electron transfer and the intercalation/deintercalation of Li^+^ [11,12]. Moreover, the path of Li^+^ diffusion is easily blocked by Fe-Li antisite defects, resulting in the diffusion coefficient of Li^+^ being much lower than the theoretical value [13]. The intrinsically low Li^+^ diffusion coefficient (~10^−14^ cm^2^·s^−1^) and electronic conductivity (~10^−10^ S·cm^−1^) cause serious capacity attenuation in the LiFePO_4_ battery in high-speed (≥5 C) charging and discharging [14,15].

Some methods have been proposed to improve the carrier conduction of cathodes, such as high-valence metal ion doping [16,17,18], high-conductivity coating [19], morphology optimization, and nanocrystallization [20,21]. High-valence metal ions usually occupy the Fe site in the LiFePO_4_ lattice, forcing defects in the lattice to balance the charge. This lattice distortion reduces the bandgap and improves the electronic conductivity of LiFePO_4_ in some cases [22]. The types and concentrations of metal ions have been widely studied. The coatings on LiFePO_4_ particles with high-conductivity materials can significantly enhance the electronic conductivity in the cathode. The high-conductivity film can be made of conductive carbon, metals, or some of their compounds (e.g., Cu [23], NiP [24], and AlF_3_ [25]), as well as conductive polymers (e.g., PEDOT [26], PPY [27], and poly[Ni(CH_3_-salen)] [28]). Conductive coatings not only enhance the conductivity of the cathode but also mechanically protect the active particles, inhibiting the dissolution of metal ions in the active material and improving the service life of the LIBs. As one of the most feasible coating materials, conductive carbon has been used in commercial LiFePO_4_. However, the current rate and cycling performance of LiFePO_4_ cathodes still cannot meet the requirements of future EVs and energy storage devices.

Recently, some researchers have reported that N, B, F, S, and other elements were doped into carbon film or graphene to improve their conductivity [29]. The doped atoms enhanced the electronic conductivity of carbon by providing carriers and enhanced the Li^+^ diffusivity in carbon by leading to defects in the graphite structure. Zhao et al. [30] proposed a pyrolysis method to prepare anode particles coated with N-doped carbon films. The conductivity and structural stability of the anode were greatly improved. Suo et al. [31] also found that N-doped carbon can improve the conductivity of the electrode and achieve a better rate performance. Ren et al. [32] introduced N, B, and F atoms into carbon film via pyrolysis of ionic liquid and observed excellent electrochemical performance on the LiFePO_4_/C-N/B/F cathode.

However, it should be noted that the preparation of the active particles coated by N-doped carbon films is complex in the existing research reports, the processes including the separate preparation of doped carbon films and active particles and also combinations of them. Moreover, some expensive reactants were usually selected as N sources in the existing research reports. Therefore, with the aim of promoting the large-scale production of high-performance cathodes, it is necessary to simplify the preparation of the LiFePO_4_@N-doped C composites and reduce the cost.

This work presents a convenient one-pot method for preparing LiFePO_4_/N-doped C with core–shell structure composites. Cetyltrimethylammonium bromide (CTAB) was used as a N source and surface modifier, attached to the LiFePO_4_ particle and carbon microsphere surface during the solvothermal reaction and then pyrolyzed and doped into the carbon during calcination. The effects of N-doping on the chemical composition and structure of LiFePO_4_/C composites were analyzed by XRD, SEM, inter alia. The electrochemical properties of these composites were measured by electrochemical tests. As expected, N-doping and the establishment of core–shell structures synergistically enhance the capacity and cycling performance of the LiFePO_4_/N-doped C cathode.

## 2. Materials and Methods

### 2.1. Preparation of the LiFePO_4_/N-Doped C Composites

**Figure 1:**
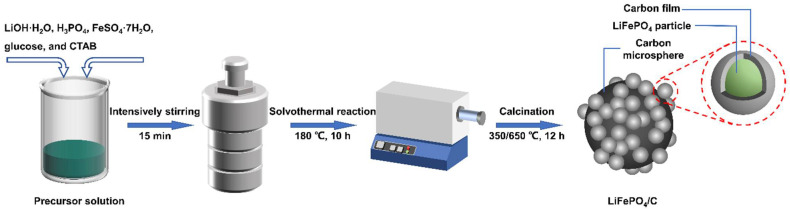
Schematic diagram of the synthesis of LiFePO4/N-doped C composites with core–shell structures.

Figure 1 shows a schematic diagram for preparing the LiFePO_4_/N-doped C composites (LFP/C-N). The preparation method is based on reports from the literature [33]. Firstly, glucose (C_6_H_12_O_6_·H_2_O), as a carbon source, and cetyltrimethylammonium bromide (CTAB, 3 mmol) were added to a mixture of deionized water and ethylene glycol (4:1, vol%). Then, the glucose solution, with a concentration of 0.3 mol·L^−1^, was obtained after vigorous stirring. The LiOH·H_2_O (18 mmol) was dissolved in the glucose solution (20 mL), and then the H_3_PO_4_ (0.2 mL, 85%) was added to form a white suspension (pH = 5.0). In addition, the FeSO_4_·7H_2_O (5.82 mmol) was dissolved in the above glucose solution (10 mL). After that, FeSO_4_ solution was dropped into the white suspension, with intensive stirring. After obtaining the green suspension, the solution was stirred for another 15 min. Twelve milliliters of the resulting precursor was added into a Teflon-lined stainless steel reactor (with a volume of 25 mL) for solvothermal synthesis at 180 °C for 10 h. The obtained powder was washed three times with deionized water and then dried at 60 °C overnight. Finally, the powder was calcined successively at 350 °C for 6 h and 650 °C for 6 h at a heating rate of 3 °C·min^−1^ in a tube furnace with an H_2_/Ar (5/95, vol) atmosphere. As a result, the LFP/C-N were obtained. The preparation of the LFP/C is the same as the LFP/C-N, but the CTAB was not used. For further comparison, the sample obtained when neither glucose nor CTAB was added was named “LFP”, and the sample obtained when CTAB was added without glucose was named “LFP-N”, as shown in Table 1. Glucose (20 wt.%) was added to the LFP and the LFP-N before calcination to form a carbon film on the LiFePO_4_ particles.

### 2.2. Structure and Morphology Characterizations

The structures of the samples were detected by X-ray diffraction (XRD, Bruker, D8 ADVANCE) with Cu-Kα radiation (λ = 0.15406 nm) in the 2*θ* range of 10°–70° at a scan speed of 12° min^−1^. Thermal gravimetric analysis (TGA) measurements with an air atmosphere and a heating rate of 10 °C·min^−1^ were carried out using an integrated thermal analyzer (NETZSCH, STA 449C). The morphology was observed with a field emission scanning electron microscope (FE-SEM, TESCAN, MALA3 LMH) and transmission electron microscope (TEM, JEOL, JEM-2100). The distribution of nitrogen in the sample particles was characterized by energy dispersive spectroscopy (EDS). X-ray photoelectron spectroscopy (XPS) analysis was performed on a photoelectron spectrometer (THERMO FISHER, ESCALAB Xi^+^). Raman spectra were collected in the shift range of 200–3000 cm^−1^ using a laser Raman spectrometer (HORIBA) with a 532 nm excitation laser.

### 2.3. Electrochemical Characterization

All samples were made into CR2032 coin-type cells. The LiFePO_4_/C powder was mixed with a conductive agent (acetylene black) and a binder (polyvinylidene fluoride, previously dissolved in N-methyl pyrrolidone) at a mass ratio of 8:1:1 [32]. The slurry obtained was coated on clean aluminum foil with a mass load of 1.9–2.1 mg·cm^−2^ and then dried in an infrared oven at 100 °C for 12 h. The cathode piece obtained was cut into a circular sheet with a diameter of 12 mm. Lithium foil and Celgard 2400 (polypropylene) were used as counter-electrodes and separators, respectively. The coin cells were assembled inside a glove box with LiPF_6_ (1 mol·L^−1^) dissolved in a mixture of EC: EMC (1:1, vol%) as the electrolyte. Then, galvanostatic charge and discharge tests in a voltage range of 2.5–4.2 V were carried out in a battery testing system (LAND, CT2001A). In the calculation of discharge capacity, carbon components in the samples were deducted. Cyclic voltammetry curves (CVs), in a voltage range of 2.5–4.2 V, and electrochemical impedance spectroscopy (EIS), at a frequency of from 0.01 Hz to 100 kHz and voltage amplitude of 5 mV, were measured using an electrochemical workstation (CHI660E).

## 3. Results and Discussion

### 3.1. Analysis of Structure and Morphology

**Figure 2:**
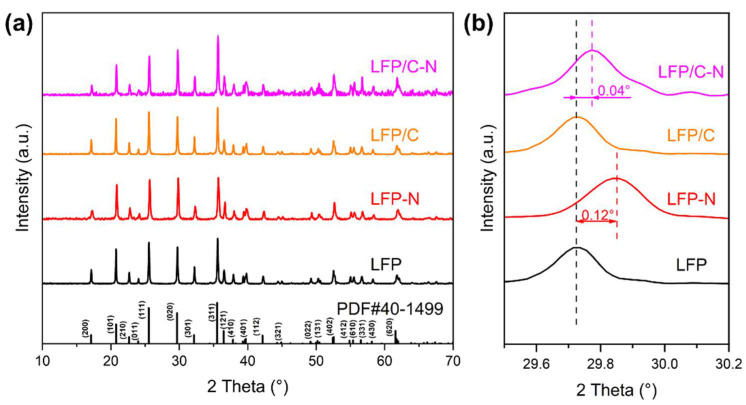
(**a**) XRD patterns and (**b**) their magnification in the range of 29.5°–30.2° of the LFP, the LFP-N, the LFP/C, and the LFP/C-N.

Figure 2a shows the XRD patterns of the LFP, the LFP-N, the LFP/C, and the LFP/C-N samples. All the sample matched well with the standard PDF card (JCPDS no. 40-1499) and no additional phases were found, indicating that there was no effect of CTAB on the reaction type of solvothermal crystallizing. As an anisotropic electrode material, the diffusion of Li^+^ in LiFePO_4_ is preferentially carried out in the direction perpendicular to (020). Therefore, the Li^+^ diffusion in the electrode and electrochemical kinetics are improved with the increase in the proportion of the (020) crystal plane occupying the surface of LiFePO_4_ particles [34,35]. The increase in the relative intensity of the (020) peak indicates that the (020) plane became relatively rich on the grain. The increase in the relative intensity of the (020) peak reflects the richer (020) plane on the grain. There is a strong (020) peak in the LFP-N and the LFP/C-N, indicating that the addition of CTAB is conducive to the emergence of the active surface.

The amplification of the XRD patterns in the range of 29.5°–30.2° are shown in Figure 2b. The diffraction peaks of the LFP-N and the LFP/C-N are shifted to the right by 0.12° and 0.04°, respectively, while those of the LFP and the LFP/C are consistent, indicating that the addition of glucose did not change the spacing of each crystal plane of LiFePO_4_. The addition of CTAB shrank the spacing of each crystal plane of LiFePO_4_, which is not conducive to the Li-ion diffusion between crystal planes. However, the addition of glucose inhibited this shrinkage. Another important reason for the increase in crystal plane spacing is the formation of defects in the crystal. Therefore, without considering the doping of the LiFePO_4_ phase, a relatively smaller cell volume indicates a relatively higher crystallinity of LiFePO_4_, which helps to improve the cycle stability of the LIBs [36].

**Figure 3:**
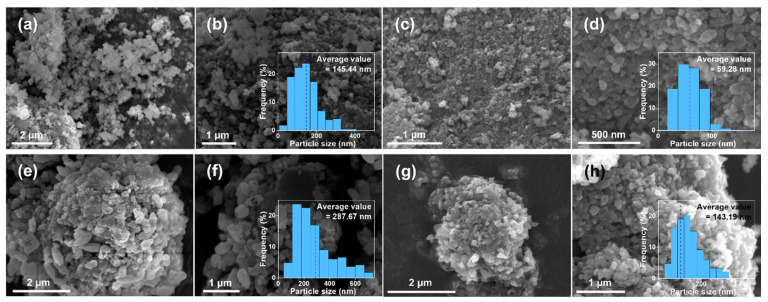
SEM images and frequency distribution histogram of the LiFePO_4_ primary particle sizes of the LFP (**a**,**b**), the LFP-N (**c**,**d**), the LFP/C (**e**,**f**), and the LFP/C-N (**g**,**h**).

The micromorphology of the composites was characterized by SEM, as shown in Figure 3. The average particle sizes of the LFP and the LFP-N were 145 nm and 59 nm, respectively (Figure 3a–d). The more uniform particle shape and the narrower particle size distribution indicates that CTAB significantly regulated the growth of LiFePO_4_ particles. In the solvothermal process, glucose formed carbon microspheres through hydrothermal carbonization, and then the LiFePO_4_ particles were attached to it to form composites with a diameter of 2–5 μm, as shown in Figure 3e–h. The particle size distribution of LiFePO_4_ primary particles in the LFP/C was uneven, and some “rice particles” with a length and width of 400–700 nm and 200–400 nm, respectively, were found. Figure 3f shows the particle size frequency distribution histogram of the LFP/C, and the average particle size is 288 nm. The larger LiFePO_4_ particles were more difficult to attach to the carbon microspheres due to the smaller specific surface area, so there were more exposed carbon microspheres in the LFP/C, as shown in Figure 3f. With the same core–shell structure, the particle size of LiFePO_4_ primary particles in the LFP/C-N decreased markedly. The average particle size of 143 nm (Figure 3h) and the particle size distribution close to a normal distribution indicated that the “aggregation growth” and “Ostwald ripening” of the LiFePO_4_ nanocrystals were effectively inhibited in the growth process [37]. During the crystallization of LiFePO_4_, CTAB (soft template) was combined with the specific crystal surface of nanocrystals, which affected the growth rate of different crystal planes in the growth process. As a result, change in the particle size and proportion of some crystal surfaces occurred [38,39,40,41], which is consistent with the XRD patterns.

**Figure 4:**
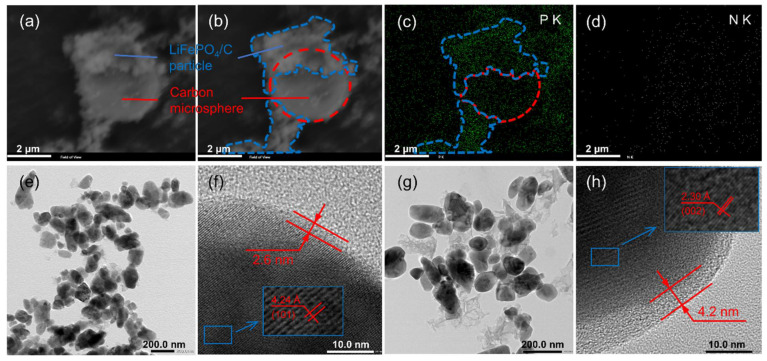
(**a**) SEM image of EDS area in the LFP/C-N and (**b**) schematic diagram of carbon microspheres and LiFePO_4_ particles. EDS mapping images of P (**c**) and N (**d**) in the LFP/C-N. TEM image of the LFP/C (**e**,**f**) and the LFP/C-N (**g**,**h**).

In order to prove that N atoms were successfully doped into the carbon film and/or carbon microspheres, SEM images of bare carbon spheres in the LFP/C-N are shown in Figure 4a,b. The carbon microspheres (red dotted lines) are partially coated by LiFePO_4_/C particle agglomerations (blue dashed lines). In the LFP/C-N, there is an apparent P element density difference between the two areas according to the EDS mapping images of the P element (Figure 4c), which proves the correctness of the above description. N elements were evenly found in LiFePO_4_/C particle and carbon microsphere areas (Figure 4d), indicating synchronous N-doping in the carbon film on the surface of LiFePO_4_ primary particles and carbon microspheres.

TEM images of the LFP/C and the LFP/C-N are shown in Figure 4e–h. The particle shape and size of all the samples in the low-magnification images (Figure 4e,g) are consistent with their SEM images. The carbon film on the LiFePO_4_ primary particle surface can be found in the high-magnification image, as shown in Figure 4f,h. The carbon film can effectively improve the charge transfer kinetics between active particles. The thickness of the carbon film in the LFP/C and the LFP/C-N is about 2.6 nm and 4.2 nm, respectively. The uniformity of the carbon film was not affected by CTAB. The thicker carbon film in the LFP/C-N was due to the residual carbon formed by CTAB attached to the surface of LiFePO_4_ primary particles.

**Figure 5:**
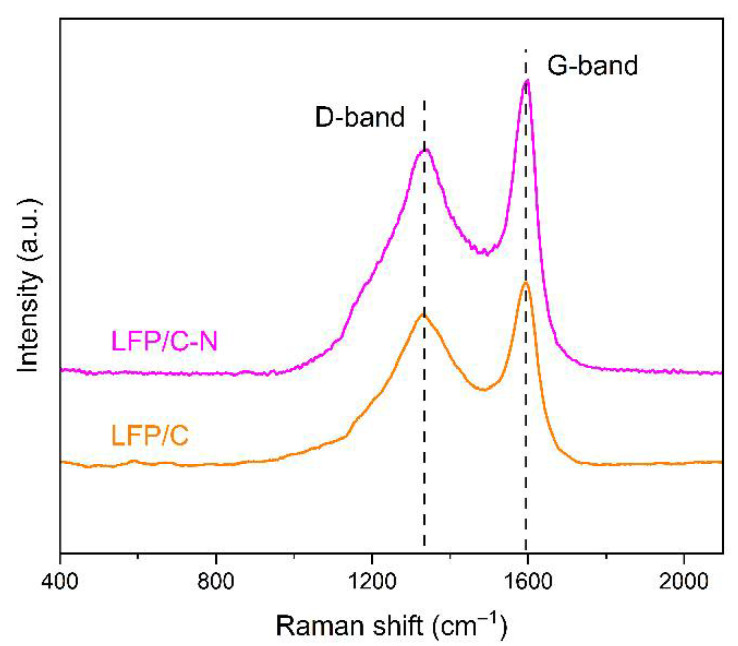
Raman spectra of the LFP/C and the LFP/C-N.

The Raman spectra of the LFP/C and the LFP/C-N are shown in Figure 5, in which the response peaks at 1350 cm^−1^ and 1590 cm^−1^ are the characterizations of carbon. The peak at 1350 cm^−1^ corresponds to the disordered state in sp^3^ hybrid carbon (D-band), i.e., amorphous carbon; the peak at 1590 cm^−1^ corresponds to the G-band of relative motion of two adjacent carbon atoms in sp^2^ graphite. The relative intensity of these two peaks can reflect the degree of graphitization of carbon to a certain extent. The lower the peak intensity ratio *I*_D_/*I*_G_ (sp^3^/sp^2^), the higher the graphitization degree in carbon. The values of *I*_D_/*I*_G_ of the LFP/C and the LFP/C-N are 0.82 and 0.77, respectively, indicating that the carbon in the LFP/C-N possesses a higher degree of graphitization than that of the LFP/C. The electronic conductivity of carbon increases with the increase in graphitization degree in carbon, which is conducive to the rate performance of the LiFePO_4_/C composite cathodes [42].

**Figure 6:**
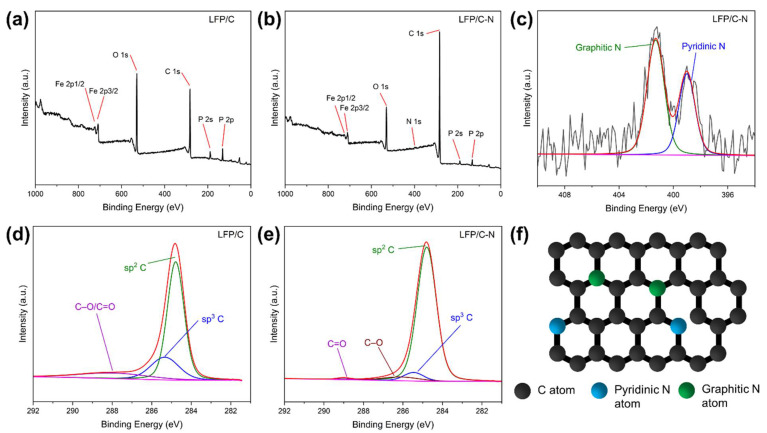
XPS spectra and the high-resolution spectra in the region of C 1s for the LFP/C (**a**,**d**) and the LFP/C-N (**b**,**e**), and the high-resolution XPS spectra in the region of N 1s for the LFP/C-N (**c**). The red line is the sum of the sub-peaks (blue, green, etc.). (**f**) Schematic structure of N-doped carbon.

The XPS spectra of the LFP/C and the LFP/C-N were measured to confirm the element composition and chemical state on the surface of samples. The peak areas in XPS reflect the atomic contents. Figure 6a,b shows the XPS spectra of the LFP/C and the LFP/C-N, in which the peak intensity of C 1s in the LFP/C-N is higher than that of the LFP/C. The LFP/C-N possesses a higher carbon content in the sample and is more completely wrapped by carbon, which is consistent with the TEM images. The overall conductivity of the cathode and the ensuing rate performance can be benefited by the formation of completely carbon film-wrapped LiFePO_4_ particles. The high-resolution XPS spectra in the region of C 1s for the LFP/C-N and the LFP/C are shown in Figure 6d,e. The C 1s peaks of the LFP/C-N and the LFP/C are mainly composed of sp^2^ and sp^3^ peaks, corresponding to graphitic carbon and amorphous carbon, respectively. In addition, C=O and C–O are detected in the high-resolution XPS spectrum of the LFP/C-N. In the main peaks of C 1s, the sp^2^ peak of the LFP/C-N occupies a larger area (94%) than that of the LFP/C (78%), indicating that there is higher sp^2^ graphite content in the LFP/C-N, which is consistent with the Raman spectra of the LFP/C and the LFP/C-N. The peak of N 1s is detected near 400 eV in the XPS spectrum of the LFP/C-N, and the N atoms come from the pyrolysis of CTAB [43,44]. The high-resolution XPS spectrum in the region of N 1s for the LFP/C-N is shown in Figure 6c. The N 1s peaks consist of two sub-peaks, namely, the pyridinic N at 398.9 eV and the graphitic N at 401.3 eV [45]. In the XPS of N 1s, the contents of graphitic N and pyridinic N are 63.5 at.% and 36.5 at.%, respectively. A pyridinic N is connected by two C atoms with lone-pair electrons, which can be oxidized; a graphitic N is connected by three carbon atoms in the graphite structure, as shown in Figure 6f. N-doping in carbon coatings can broaden the energy bandgap, adjusting the electronic structure and enhancing the density of available carriers and the consequent electronic conductivity of carbon. In addition, pyridinic N can destroy the ordered structure of graphite and produce defects to improve Li^+^ diffusion in carbon materials [29].

### 3.2. Electrochemical Properties

**Figure 7:**
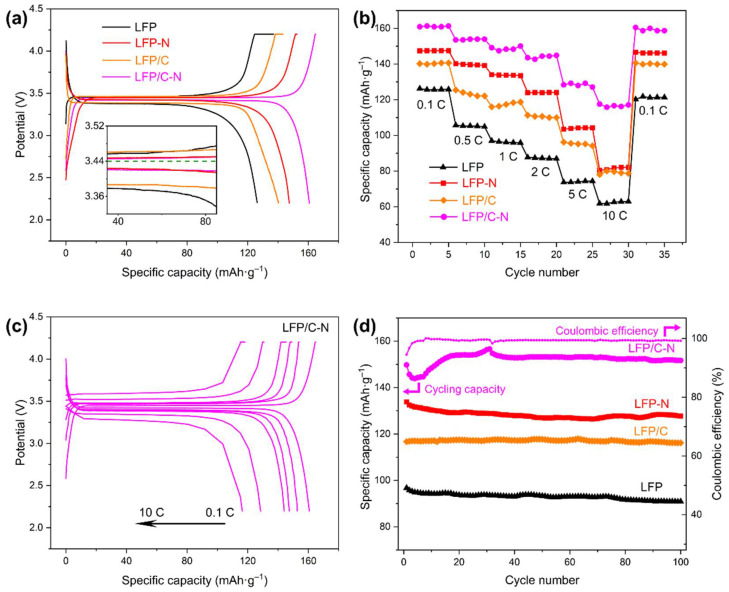
(**a**) Initial charge/discharge curves of the samples from the LFP to the LFP/C-N at 0.1 C. (**b**) Discharge capability of the samples from the LFP to the LFP/C-N at a different rate. (**c**) Charge/discharge curves of the LFP/C-N at a different rate. (**d**) Cycling performance curves of the samples at 1 C.

The initial charge/discharge voltage profiles at 0.1 C of the LFP, the LFP-N, the LFP/C, and the LFP/C-N were measured. The carbon content in the samples was obtained by TGA (Table 1) and was deducted in the calculation of the charge/discharge capacity. Figure 7a shows the initial specific discharge capacities of 126.2 mAh·g^−1^ (LFP), 140.3 mAh·g^−1^ (LFP/C), 147.4 mAh·g^−1^ (LFP-N), and 160.7 mAh·g^−1^ (LFP/C-N), respectively. Both the core–shell structure and the N-doping enhanced the charge/discharge capacities of the cathodes, but the N-doping played a more significant role. The pyridinic N atoms in the LFP/C-N destroyed the ordered structure of carbon, produced structural defects, and then improved the diffusion of Li-ion in the cathodes.

The specific discharge capacities of the samples at different rates are shown in Figure 7b. The LFP/C-N delivered a specific discharge capacity of 128.4 mAh·g^−1^ at 5 C with a capacity retention of 76.88%, and even 115.8 mAh·g^−1^ at 10 C, which is higher than that of the LiFePO_4_/(N-doped C) described in similar research reports, as shown in Table 2. Furthermore, the preparation method of the LiFePO_4_/(N-doped C) in this work is more convenient than those shown in Table 2. Figure 7c shows the charge and discharge voltage profiles of the LFP/C-N at different rates. A flat voltage platform is still found at the high rate of 5 C and 10 C. The satisfactory rate performance in the LFP/C-N was attributed to the effect of the N-doped core–shell structure on the overall electronic and Li-ion conductivity of the LiFePO_4_/C cathodes. The N-doping in carbon both enhanced the electronic conductivity and the Li-ion diffusivity in carbon and improved the charge transfer on the surface of active particles at high charge and discharge rates.

To investigate the cycling performances of LFP/C-N, the discharge specific capacities of these samples in 100 cycle charge/discharge at 1 C were measured, as shown in Figure 7d. The cycling capacity retentions were 94.01% (LFP), 99.55% (LFP/C), 95.43% (LFP-N), and 101.29% (LFP/C-N), respectively. The excellent cycling performance of the LFP/C and the LFP/C-N was mainly attributed to the core–shell structure with the carbon microspheres as the core. The carbon microspheres established a conductive medium between active particles and inhibited the mechanical degradation of the cathode materials during cycling [46]. The specific discharge capacity of LFP/C-N increased in the early stage of the cycling test. This is a common phenomenon in batteries, especially in those assembled in the laboratory. As the cycle progresses, the wettability of the active material gradually increased, that is, the activation of the LiFePO_4_ particles led to the gradual increase in the capacity of the coin cell. After the activation was completed in the first 30 charge/discharge cycles, the specific discharge capacity was also stable.

**Table 2:** The discharge capacity of LiFePO_4_/(N-doped C) in similar research reports.

Coating Materials	Preparation Method	N Source	Discharge Capacity (mAh·g^−1^)	Ref.
N-doped C	High-temperature, solid-state method and subsequent calcination with addition of N and C sources	Ionic liquid1-butyl-3-methylimidazolium dicyanamide	127.1 mAh·g^−1^ at 0.1 C	[47]
N-doped 3D graphene	Hydrothermal synthesis based on prepared N-doped graphene	Melamine	125 mAh·g^−1^ at 5 C	[48]
N-doped C and TiO_2_	Coating commercial LiFePO_4_ with TiO_2_ and C sources by wet chemical method	Polydopamine	124 mAh·g^−1^ at 2 C	[49]
N-doped C	Solvothermal synthesis of LiFePO_4_ powder and subsequent calcination with addition of N and C sources	N-methyl-N-propylpyrrolidinium bis(trifluoromethyl sulfonyl)imide	102.8 mAh·g^−1^ at 5 C	[50]
N-doped C	One-pot solvothermal method	CTAB	128.4 mAh·g^−1^ at 5 C	This work

**Figure 8:**
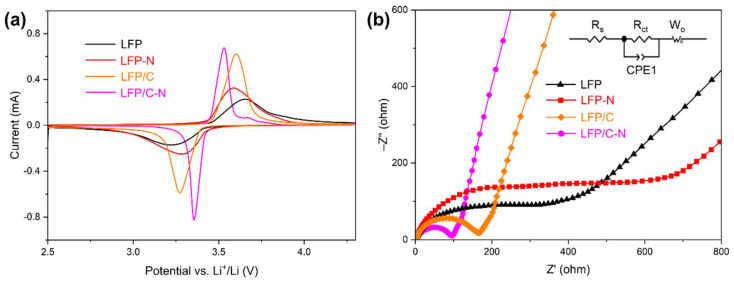
(**a**) Cyclic voltammetry curves of the samples from the LFP to the LFP/C-N between 2.5 and 4.6 V (vs. Li^+^/Li) at a rate of 0.1 mV·s^−1^. (**b**) Electrochemical impedance spectra of the samples from the LFP to the LFP/C-N, and equivalent circuit model for fitting the experimental data of EIS.

Figure 8a shows the CV curves of the LFP, the LFP-N, the LFP/C, and the LFP/C-N that were measured at a scan rate of 0.1 mV·s^−1^. All the samples reflect a pair of Fe^2+^/Fe^3+^ redox peaks near 3.3 V and 3.6 V, corresponding to the Li^+^ intercalation and deintercalation reaction in LiFePO_4_/FePO_4_ [51]. The symmetry of the peaks of the CV curve reflects the reversibility of the electrochemical process. The CV curves of each sample show a good symmetry, that is, the cathode materials possess a good cycling performance, which is consistent with the results of the cycling test. The LFP/C and the LFP/C-N with core–shell structures show sharper redox peaks, indicating fast electrochemical kinetics and a charge transfer. The LFP/C-N delivered the lowest redox potential difference of 176 mV, indicating the smallest polarization degree and the highest cycling reversibility in the LFP/C-N.

As shown in Figure 8b, the EIS of the samples was measured to investigate the kinetic characteristics of the electrochemical process in the cathodes. The EIS was composed of the charge transfer impedance corresponding to the semicircle in the high-frequency region, and the diffusion impedance corresponding to the oblique line in the low-frequency region. In the equivalent circuit, *R*_s_ is the ohmic resistance, which represents the resistance of the electrolyte and electrode material and corresponding to the intercept of the curve on the *Z*’ axis; *R*_ct_ refers to the charge transfer resistance between the active material (LiFePO_4_) and the electrolyte; CPE1 refers to the constant phase angle element; *W*_o_ is the Warburg resistance, corresponding to the Li^+^ diffusion in the active material. Table 1 shows the *R*_s_ and *R*_ct_ obtained by fitting EIS and the equivalent circuit. The *R*_ct_ of LFP/C (137 Ω) and the LFP/C-N (83.1 Ω) with core–shell structure were lower than those of the LFP and the LFP-N, indicating a higher electronic conductivity at the electrode-electrolyte interface in the LFP/C and the LFP/C-N. The charge transfer on the surface of LFP/C-N particles was further promoted by the uniform N-doped carbon film coating. The *R*_ct_ of the LFP-N was higher than that of the LFP, while the *R*_ct_ of the LFP/C-N was lower than that of the LFP/C, indicating a synergistic effect on the reduction in charge transfer resistance between the core–shell structure and N-doping.

The Li^+^ diffusion coefficient (*D*) in the active particles can be calculated using the following equation [52,53]:

(1) $$D=\frac{R^{2}T^{2}}{2S^{2}n^{4}F^{4}C^{2}\sigma^{2}}$$

where *D* (cm^2^·s^−1^) is the Li^+^ diffusion coefficient in the active particles, *R* is the gas constant (8.314 J mol^−1^·K^−1^), *T* (K) is the temperature, *S* (cm^2^) is the surface area of the cathode (1.131 cm^2^), *n* is the number of electrons per molecule during oxidization, *F* is the Faraday constant (96,486 C·mol^−1^), *C* is the concentration of the Li^+^ (7.69 × 10^−3^ mol·mL^−1^), and σ is the Warburg factor, which is the slope between *Z’* in the low-frequency region and the reciprocal square root of the frequency (*ω*^−1/2^), as expressed in the following equation:

(2) $$Z^{\prime }=R_{s}+R_{\mathrm{ct}}+\sigma \omega^{-1/2}$$

The electrochemical kinetic parameters of the samples are also shown in Table 1. There is little difference in the diffusion of Li-ion coefficient among samples, indicating that the addition of CTAB and/or glucose had no significant effect on the diffusion of Li-ion in the LiFePO_4_ particles, but only changed the charge transfer on the LiFePO_4_/C particles.

## 4. Conclusions

In this work, LiFePO_4_/C with N-doped core–shell structure composite cathodes were synthesized by a convenient solvothermal method with the assistance of CTAB and glucose. The addition of CTAB improved the crystallinity of LiFePO_4_ and reduced the size of LiFePO_4_ primary particles from 288 nm to 143 nm. The N-doping in the carbon microspheres improved the degree of graphitization of the carbon in the LFP/C-N and existed in carbon in the form of pyridinic N and graphitic N. The electrochemical measurement results showed that the LFP/C-N had better electrochemical performance than the other samples. The LFP/C-N delivered a specific discharge capacity of 160.7 mAh·g^−1^ and 128.4 mAh·g^−1^ at 0.1 C and 5 C, respectively. There was no capacity attenuation in the LFP/C-N after the 100 cycles of charge/discharge at 1 C. The excellent capacity performance of the LFP/C-N is mainly attributed to the N-doping in carbon, while the excellent cycling performance is mainly attributed to the special core–shell structure. It is worth noting that there is a synergistic effect between N-doping and core–shell structure on the reduction in charge transfer impedance of the LiFePO_4_/C cathode.

## Figures and Tables

**Table 1:** Synthesis conditions, carbon content, and electrochemical kinetic parameters of the LFP, the LFP-N, the LFP/C, and the LFP/C-N.

Sample Name	Additives in Synthesis (mol·L^−1^)		Carbon Content (%)	Ohmic Resistance (*R*_s_, Ω)	Charge Transfer Resistance (*R*_ct_, Ω)	Li^+^ Diffusion Coefficient (*D*, cm^2^ s^−1^)
	Glucose	CTAB				
LFP	0	0	4.71	1.32	269	7.28 × 10^−14^
LFP-N	0	0.1	6.71	1.29	438	5.95 × 10^−14^
LFP/C	0.3	0	18.46	1.84	137	4.90 × 10^−14^
LFP/C-N	0.3	0.1	16.85	2.09	83.1	6.35 × 10^−14^

## Data Availability

The data presented in this study are available on request from the corresponding author after obtaining permission from an authorized person.
